# Epigenetic Activation of Wnt/β-Catenin Signaling in NAFLD-Associated Hepatocarcinogenesis

**DOI:** 10.3390/cancers8080076

**Published:** 2016-08-20

**Authors:** Yuan Tian, Myth T.S. Mok, Pengyuan Yang, Alfred S.L. Cheng

**Affiliations:** 1 Key Laboratory of Infection and Immunity, Institute of Biophysics, Chinese Academy of Sciences, Beijing 100101, China; teresaty1987@163.com; 2 School of Biomedical Sciences, The Chinese University of Hong Kong, Hong Kong, China; mythmok@mythmok.com; 3 Shenzhen Research Institute, The Chinese University of Hong Kong, Shenzhen 518057, China; 4 State Key Laboratory of Digestive Disease and Institute of Digestive Disease, The Chinese University of Hong Kong, Hong Kong, China

**Keywords:** non-alcoholic fatty liver disease, hepatocellular carcinoma, Wnt, β-catenin, epigenetics, DNA methylation, histone modification, microRNA, HDAC8

## Abstract

Non-alcoholic fatty liver disease (NAFLD), characterized by fat accumulation in liver, is closely associated with central obesity, over-nutrition and other features of metabolic syndrome, which elevate the risk of developing hepatocellular carcinoma (HCC). The Wnt/β-catenin signaling pathway plays a significant role in the physiology and pathology of liver. Up to half of HCC patients have activation of Wnt/β-catenin signaling. However, the mutation frequencies of *CTNNB1* (encoding β-catenin protein) or other antagonists targeting Wnt/β-catenin signaling are low in HCC patients, suggesting that genetic mutations are not the major factor driving abnormal β-catenin activities in HCC. Emerging evidence has demonstrated that obesity-induced metabolic pathways can deregulate chromatin modifiers such as histone deacetylase 8 to trigger undesired global epigenetic changes, thereby modifying gene expression program which contributes to oncogenic signaling. This review focuses on the aberrant epigenetic activation of Wnt/β-catenin in the development of NAFLD-associated HCC. A deeper understanding of the molecular mechanisms underlying such deregulation may shed light on the identification of novel druggable epigenetic targets for the prevention and/or treatment of HCC in obese and diabetic patients.

## 1. Epigenetics in NAFLD-HCC

Hepatocellular carcinoma (HCC) is one of the most common malignancies in the world and the second leading cause of cancer-related mortality [1]. In the past decade, the incidence of HCC has been increasing in almost all countries. Although many risk factors of HCC are well documented, such as infection of hepatitis B virus (HBV) and hepatitis C virus (HCV), obesity and type 2 diabetes, the latter two factors have emerged as the major factors responsible for the recent increase of HCC incidence [2]. Non-alcoholic fatty liver disease (NAFLD), characterized by fat accumulation in liver, is a wide spectrum of liver diseases ranging from simple fatty liver to non-alcoholic steatohepatitis (NASH), which may progress further to end-stage liver diseases like cirrhosis and HCC [3]. NAFLD is closely associated with central obesity, over-nutrition, insulin resistance and other features of metabolic syndrome [4]. It seems likely that the growing epidemic of NAFLD-associated HCC can be attributed to westernized and over-nutrition lifestyles, particularly in Asian populations [5,6].

Epigenetics refers to chromatin modifications exclusive of alterations in nucleotide sequences. It mainly includes DNA methylation, histone modifications and microRNAs (miRNAs), which can, respectively, activate or silence gene transcription (or act post-transcriptionally in the case of miRNAs), leading to diverse functional effects. Indeed, complex roles have been reported for a variety of chromatin modifiers, including but not limited to DNA methyltransferases (DNMTs), histone methyltransferases (HMTs), histone acetyltransferases (HATs) and histone deacetylases (HDACs) [7]. It is now evident that epigenetics plays crucial roles in many human diseases particularly cancer [8], especially in HCCs [9,10,11,12,13,14]. However, the roles of epigenetic regulation in metabolic abnormalities still remain elusive [15].

Although genomic study of NAFLD has been an established area of research for some time, the relevance of epigenomic factors such as DNA methylation in NAFLD development has only become apparent in recent years [16]. For instance, Pogribny and colleagues identified that mice fed with lipogenic methyl-deficient diet suffered from liver injuries resembling human NASH, in which livers displayed altered expression of the DNA methyltransferases DNMT1 and DNMT3A, less cytosine methylation at genomic and repetitive sequences, and abnormal histone modifications [17]. Concordantly, other studies demonstrated that many NAFLD candidate genes are aberrantly methylated in obesity or type 2 diabetes, and displayed altered expression in HCCs [18,19]. In addition, Relton et al. demonstrated some genes that were significantly correlated with variation in body size displayed hypermethylation in gene promoters or reduced gene expression in HCC [20]. Among them, *ALOX25* and *IRF5* are of interest because they are consistently hypermethylated in obesity and HCC samples [18,19].

Recently, altered expression and activities of certain HDACs/HATs have been linked to deregulated histone acetylation and gene expression in NAFLD, leading to abnormal metabolism and cellular transformation. Several HDACs have been shown to be critical modulators in the pathophysiology of NAFLD. Among them, HDAC3 is the most well-studied epigenetic modifier that can govern both circadian metabolic activity and hepatic lipid homeostasis [21,22,23]. Additionally, HDAC3 is frequently up-regulated in liver cancers, which make it a critical linkage between NAFLD and HCC [15].

MiRNAs have emerged as key regulators of metabolism [24]. The metabolic miRNAs, including miR-33, miR-103, miR-107 and miR-143, play pivotal roles in controlling the metabolism and homeostasis of insulin, glucose, cholesterol and lipid in vivo [25,26,27]. It has become clear that alterations in the expression of miRNAs contribute to the pathogenesis of most human cancers [28,29,30]. A number of differentially-expressed miRNAs that function in hepatic cholesterol and fatty acid homeostasis have been shown to contribute to the pathogenesis of HCC in NAFLD [15].

## 2. Wnt/β-Catenin Signaling and Epigenetics

The canonical or Wnt/β-catenin pathway is the best characterized Wnt pathway. Up to half of HCC patients have activation of Wnt/β-catenin signaling pathway [31,32,33]. When Wnt signaling is suppressed, generally due to a combination of lack of Wnt ligands and a prevalence of Wnt antagonists, β-catenin is sequentially phosphorylated by CK1 and GSK3β [34]. Upon phosphorylation, β-catenin is recognized by β-transducin repeat-containing protein for ubiquitination and degradation [35]. Alternatively, activation of Wnt signaling via interaction of a Wnt ligand with the Frizzle/LRP co-receptor complex halts the β-catenin degradation process, resulting in β-catenin accumulation in the cytoplasm and subsequent translocation into the nucleus, which then partners with the nuclear transcription complex TCF/LEF to increase the expression of a range of downstream targets to promote liver cancer formation [36].

An increasing body of evidence indicates that regulators controlling Wnt/β-catenin signaling are frequently dysregulated in human cancers owing to genetic and epigenetic defects [37]. Mutations of pathway components including *APC*, *AXIN1/2* and *CTNNB1* (encoding β-catenin) are common in HCC. These tumor-causative mutations lead to inappropriate stabilization of β-catenin, which persistently activates target genes associated with cell proliferation and transformation, such as cell cycle drivers cyclin D1 (*CCND1*) and c-Myc [38,39]. Alternatively, functional loss of Wnt inhibitors by epigenetic silencing, through either DNA methylation or histone modification, has also been recently reported to contribute to the aberrant activation of Wnt/β-catenin signaling in tumors. Different classes of epigenetically deregulated Wnt antagonists include extracellular Wnt inhibitors (SFRP1-5, WIF1 and DKK1-3), cytosolic Wnt inhibitors (DACT1-3, AXIN2, APC), nuclear factors (SOX7, 17), Wnt non-transforming ligands (Wnt5A, 7A, 9A), and epithelial adhesion molecules (CDH1) [40]. The growing list of epigenetically silenced Wnt antagonists in various human cancers suggests an important role for epigenetic regulation of Wnt/β-catenin pathway in tumor initiation and progression. For examples, TCF/β-catenin binding to Wnt responsive element (WRE) can lead to histone acetylation in a CBP-dependent manner over a significant genomic distance (30 kb), suggesting that local TCF/β-catenin recruitment results in widespread chromatin modifications. In addition, histone H3 lysine 4 trimethylation (H3K4me3), a typical indicator of active gene transcription, was detected at the WRE of c-*Myc* gene, which is a known Wnt target gene in cancer cells [41].

## 3. Epigenetic Regulation of Wnt/β-Catenin Signaling in NAFLD-HCC

Activation of Wnt/β-catenin signaling can drive the expression of specific oncogenes in various human cancers. *CTNNB1*, *APC* and *AXIN* gene mutations are classic defects that trigger chronic Wnt signaling in cancers [42]. However, the frequencies of these mutations are very low in certain malignancies such as HCC, wherein *CTNNB1* mutation rate was 12%–16%, *AXIN* mutation rate was 8%–15%, and no mutation of *APC* was reported [43,44]. This evidence implicates that epigenetic deregulation may account for the abnormal Wnt/β-catenin activity in these models [45]. Indeed, in 100 human frozen liver biopsies of mild and advanced NAFLD patients, 69,247 differentially methylated CpG sites (between mild and advanced disease) with correlated expression changes were identified. In samples with advanced NAFLD, many tissue repair genes were hypomethylated and overexpressed, and genes in certain metabolic pathways, including 1-carbon metabolism, were hypermethylated and underexpressed. These findings support that epigenetic dysregulation is associated with NAFLD progression and HCC initiation [46].

### 3.1. DNA Methylation

The secreted frizzled-related protein (SFRP) family is a class of extracellular Wnt inhibitors that act at cell membrane to prevent Wnt-mediated induction of β-catenin [47]. Promoter methylation-dependent silencing of these extracellular Wnt antagonists, including *SFRP1*, *SFRP2*, *SFRP4* and *SFRP5*, correlates with constitutive activation of canonical Wnt/β-catenin signaling in liver cancers [48,49]. Among them, SFRP5 hepatic expression was recently reported to be associated with NAFLD in morbidly obese people [50], and the CpG methylation level of *SFRP5* was inversely correlated with its expression in NAFLD patients [46]. These data showed that SFRP5 is functionally important and is under epigenetic regulation in NAFLD development.

SOX17 is a nuclear protein that directly interacts with TCF/LEF to inhibit the transcription of Wnt signaling target genes. Epigenetic silencing of *SOX17* through promoter methylation is a frequent event in human cancers, and it contributes to the aberrant activation of Wnt/β-catenin signaling [51]. Moreover, SOX17 plays a key role in regulating insulin secretion, as mice lacking *Sox17* were more susceptible to high fat diet-induced hyperglycemia and diabetes [52]. Functionally, hyperinsulinaemia may affect HCC development not only through direct effects on the growth of hepatocytes, but also indirectly by increasing the production of cytokines and mitogens, enhancing fibrosis and promoting angiogenesis [53]. Taken together, down-regulation of *SOX17* via promoter methylation may promote Wnt activity and insulin secretion and thereby accelerate the progression from NAFLD to HCC.

The cytosolic Wnt antagonists, such as members of the DACT gene family (DACT2 and DACT3), can antagonize Dvl and are central components of Wnt signaling [54]. Zhang and colleagues found that *DACT2* promoter methylation was inversely correlated with *DACT2* expression, which could be restored by 5-aza-2′-deoxycytidine in HCC cell lines. Of clinical significance, reduced *DACT2* expression was significantly related to promoter hypermethylation in 28 of 62 (45.16%) HCC patients, and the expression of *DACT2* was inversely related to β-catenin expression in liver cancers [55]. Moreover hypermethylation of the *DACT2* promoter was reported in advanced NAFLD individuals compared with mild NAFLD patients [46], which is consistent with its low expression in HCCs.

Distinct from *DACT2*, silencing of *DACT3* in cancer cells is mediated by a bivalent histone modification that contains both repressive histone H3 lysine 27 trimethylation (H3K27me3) and activating H3K4me3, and such *DACT3* ablation is not associated with promoter methylation [56]. Of note, it has been reported that histone modification and promoter methylation can independently regulate gene suppression [57], which illustrates the complexity of epigenetic controls on Wnt/β-catenin signaling pathway in liver cancers.

### 3.2. Histone Modifications

Based on previous studies, histone modification plays a key role in controlling the expression of Wnt inhibitors in HCC cells. Using chromatin immunoprecipitation microarray (ChIP-Chip) analysis, Cheng and colleagues discovered a panel of Wnt pathway inhibitors whose promoters were concordantly occupied by enhancer of zeste homolog 2 (EZH2) and H3K27me3 in HCC cells [14]. EZH2 is a histone methyltransferase that catalyzes the addition of methyl groups to histone H3 at lysine 27 hence inducing gene repression. Further analyses illustrated that EZH2-mediated transcriptional suppression of these Wnt signaling antagonists allows constitutive activation of Wnt/β-catenin signaling, which contributes to EZH2-driven cellular proliferation [14].

Additionally, Ezh2 was reported to directly suppress Wnt genes to facilitate adipogenesis in mice. The adipogenesis defects in cells with enzymatically inactive Ezh2 can be rescued by expression of adipogenic transcription factors PPARγ and C/EBPα, or inhibitors of Wnt/β-catenin signaling [58]. Taken together these data indicate that EZH2 plays a critical role in the pathogenesis of NAFLD/NASH, and is closely related to the HCC progression.

Additional studies demonstrated that EZH2 and HDACs work in concert at epigenetic level to reinforce the aberrant Wnt signaling activation in HCCs. Knock-down of EZH2 reduced the occupancy of HDAC1 at the promoters of Wnt antagonists in HCC cells [14]. Treatment of obese diabetic mice with a class I selective HDAC inhibitor enhanced oxidative metabolism in adipose tissue, and reduced body weight, glucose and insulin levels, indicating improved metabolism condition [59]. Consistently, HDAC1 was up-regulated in diabetes patients [60].

Another class I HDAC, HDAC8, was also reported to physically interact with EZH2 to contribute to the activation of Wnt/β-catenin signaling during HCC development [10]. HDAC8 has been recently reported to promote insulin resistance and activate Wnt/β-catenin pathway in a NAFLD-HCC mouse model treated with high fat high carbohydrate diet. Mechanistically, HDAC8 binds to the promoter regions of Wnt antagonists (*AXIN2*, *NKD1*, *PPP2R2B* and *PRICKLE1*) and promotes their silencing, and thereby increases the expression of a β-catenin target *CCND1* that in turn induces p53/p21-mediated apoptosis and G2-M phase cell cycle arrest. Lentivirus-mediated silencing of HDAC8 in vivo was sufficient to reverse insulin resistance and reduce NAFLD-associated tumorigenicity. Furthermore, HDAC8 was directly up-regulated by the lipogenic transcription factor SREBP-1, and such positive relationship is highly consistent in dietary obesity models of NASH and HCC [10].

Taken together, these studies provide links between histone modification and Wnt/β-catenin signaling in the development of NAFLD-associated liver cancers.

### 3.3. MiRNAs

Emerging evidence suggests the role of miRNAs in the regulation of key biological properties of HCC [9,13,61]. In a recent study, a NAFLD-NASH HCC model was established by high fat diet. In this model, gross anatomical examination revealed differential hepatomegaly, and histological analysis showed different degrees and levels of steatosis, inflammatory infiltration and fibrosis in the high fat diet-treated animals compared with controls, demonstrating the progression from NAFLD to NASH. Importantly, macroscopic nodules were observed in 20% of high fat diet-fed mice after 12 months of treatment. Fifteen differentially expressed miRNAs was identified in high fat diet-treated mice with respect to controls. Among the identified miRNAs, miR-125a-5p showed up-regulation and miR-182 showed down-regulation in the progression from liver damage to liver cancer formation [62].

miR-122 is the most abundant miRNA in the adult human livers. It can bind to the 3′-UTR of *Wnt1* mRNA for suppression, as well as down-regulating the protein levels of Wnt1, β-catenin and TCF-4. It was previously shown that miR-122 was under-expressed in HCC relative to normal liver tissue, suggesting that loss of miR-122 in HCC might contribute to excess Wnt signaling [63]. There are many direct and indirect targets of miR-122 involved in liver homeostasis, and the miR-122 expression was reported to be abnormal in most liver diseases [64]. miR-122 is considered to be a tumor suppressor miRNA, and recent study showed that mice lacking miR-122 were prone to quick development of steatohepatitis, fibrosis and HCC [65]. In addition to HCC, miR-122 expression was also reported to be dysregulated in *ob/ob* mouse livers [66]. It has been demonstrated that miR-370 induces the accumulation of hepatic triglycerides through miR122, which leads to increased expression of SREBP-1c and other genes controlling lipid metabolism [67]. Another study showed that hepatocytes from miR-122-depleted mice had higher fatty acid oxidation rates and less fatty acid synthesis [68]. Similarly, miR-122 antagonist significantly improved hepatic steatosis and reduced levels of triglyceride accumulation in diet-induced obese mice. Taken together, miR-122 is a risk factor of obesity and hepatic metabolic dysfunction. Moreover, hepatic miR-122 deregulation appears to contribute to NAFLD progression towards HCC, while circulating miR-122 levels correlate with disease stages and is thus a potential biomarker of NASH-HCC progression.

miR-34a was reported to be a tumor suppressor in HCCs through regulation of Wnt/β-catenin pathway. Studies showed that ectopic miR-34a induces cell-cycle arrest and apoptosis by down-regulation of *CCND1* [69,70]. In turn, miR-34a was also found to be significantly up-regulated by the over-activation of β-catenin signaling in mouse tumors and in HCC patients [71]. In chronic hepatitis patients, serum levels of miR-34a were significantly higher than those in controls, and positively correlated with disease severity from simple steatosis to steatohepatitis [72].

Another tumor suppressor miR-145 targets insulin receptor substrate-1 (IRS-1) in the insulin-like growth factor pathway and regulates resistin-induced insulin resistance [73]. Down-regulation of miR-145 could be used to differentiate between steatosis and steatohepatitis in diet-induced NASH [74], indicating its critical role in NAFLD development. Increased miR-145 expression leads to the reduction of β-catenin protein levels, thus exhibiting its tumor suppressor function in HCC.

However, there are several miRNAs that showed opposite expression patterns in metabolic disorder and HCC. MiR-214 is a tumor suppressor miRNA that can directly or indirectly targets β-catenin to inhibit cell growth by down-regulating c-Myc, cyclin D1, TCF-1 and LEF1 in HCC [75]. However, miR-214 was found to be significantly up-regulated in *ob/ob* mouse livers compared with controls [66]. Further research is required for deeper understanding of the mechanisms underlying the contradictory functions of these miRNAs in NAFLD and HCC.

## 4. Clinical Implications

In this review, we have summarized the evidence that Wnt/β-catenin signaling is epigenetically dysregulated through multiple mechanisms in NAFLD-HCC (Table 1). Of clinical significance, some epigenetic changes are pharmacologically reversible using epigenetic agents including DNMT inhibitors (such as 5-aza) and HDAC inhibitors (such as TSA, SAHA) [76]. These epigenetic inhibitors have been reported as potential therapeutic agents with promising effects in various cancers [37]. For examples, restoration of the expression of Wnt antagonists through either DNA demethylation or histone remodeling results in the blockade of β-catenin-dependent transcription, inhibition of tumor cell proliferation, and induction of tumor cell apoptosis in HCC [10,14,55,77]; and pharmacological demethylation using 5-aza-2′-deoxycytidine leads to the demethylation and expression of SFRPs, DACT2 and WNT10B, reduction of TCF/β-catenin target genes, and apoptosis in cancer cells [55,77,78,79]. In addition, targeting noncoding RNAs that are deregulated in HCC and contribute to the tumor phenotype or tumor chemosensitivity is also a feasible approach. To date dozens of miRNAs and long noncoding RNAs (lncRNA) with specific roles in HCC development have been documented in the literature, and some of which were shown to be promising therapeutic targets. For instance, phase I and II clinical trials using anti-miR-122 oligonucleotides that target miR-122 have shown both the safety and efficacy of this approach in humans [80]. Although there is no study on the effect of these inhibitors in NAFLD progression, it is anticipated that epigenetic modulation of Wnt/β-catenin signaling is a rational approach and potentially an effective therapeutic strategy for treating Wnt- and NALFD-associated liver cancers.

## Figures and Tables

**Table 1:** Epigentic regulations of Wnt/β-catenin signaling in NAFLD-HCC.

Epigenetic Regulation	Gene Name	Epigenetic Changes	Roles in Wnt/β-Catenin	Roles in NAFLD	Roles in HCC	References
DNA methylation	*SFRP5*	Hypermethylation	Prevent ligand-receptor interactions	Down-regulated in obese people with non-alcoholic liver disease	Down-regulated in HCC patients	[46,47,48]
	*SOX17*	Hypermethylation	Interact with the nuclear transcription complex TCF/LEF	Regulate insulin secretion in mice	Down-regulated in HCC patients	[51,52]
	*DACT2*	Hypermethylation	Antagonize Dvl	Hypermethylated promoter in advanced NAFLD patients	Down-regulated in HCC patients	[54,55]
Histone modification	*EZH2*	H3K27 trimethylation	Suppress AXIN2, NKD1, PPP2R2B, PRICKLE1 and SFRP5	Up-regulated in NAFLD-HCC patients and mouse model	Up-regulated in HCC patients	[10,14,58]
	*HDAC1*	Interaction with EZH2	Suppress AXIN2, NKD1, PPP2R2B, PRICKLE1 and SFRP5	Class I selective HDAC inhibitor reduces body weight, and glucose and insulin levels in mice	Up-regulated in HCC patients	[14,59,60]
	*HDAC8*	Interaction with EZH2, H4 acetylation	Suppress AXIN2, NKD1, PPP2R2B and PRICKLE1	Up-regulated in NAFLD-HCC patients and mouse model	Up-regulated in NAFLD-HCC patients	[10]
MicroRNAs	miR-122	Down-regulation	Suppress Wnt1 activity	Increased fatty acid oxidation rates and reduced fatty acid synthesis	Down-regulated in HCC patients	[63,64,65,66,67,68]
	miR-34a	Down-regulation	Induce cyclin D1 expression	Increased at serum levels in NAFLD patients	Down-regulated in HCC patients	[69,70,71,72]
	miR-145	Down-regulation	Reduce β-catenin levels	Down-regulated in mouse model	Down-regulated in HCC patients	[73,74]
